# Supplementary material for: Pyruvate Dehydrogenase Kinase 1 inhibition mediated oxidative phosphorylation enhancement in cartilage promotes osteoarthritis progression
Source: BMC Musculoskelet Disord. 2023 Jul 20;24:597. doi: 10.1186/s12891-023-06585-6 (PMC10357736; doi:10.1186/s12891-023-06585-6)
Supplement: Supplementary file 1 — Additional file 1. [file 12891_2023_6585_MOESM1_ESM.zip › KEGG.Gsea.1653622432464/KEGG_COMPLEMENT_AND_COAGULATION_CASCADES.html]

Details for gene set KEGG\_COMPLEMENT\_AND\_COAGULATION\_CASCADES[GSEA]

|  || Dataset | 55457\_collapsed\_to\_symbols.cls.cls #OA\_versus\_normal.cls.cls #OA\_versus\_normal\_repos |
| Phenotype | cls.cls#OA\_versus\_normal\_repos |
| Upregulated in class | normal |
| GeneSet | KEGG\_COMPLEMENT\_AND\_COAGULATION\_CASCADES |
| Enrichment Score (ES) | -0.14869659 |
| Normalized Enrichment Score (NES) | -0.613441 |
| Nominal p-value | 0.9918434 |
| FDR q-value | 1.0 |
| FWER p-Value | 1.0 |
Table: GSEA Results Summary

  

Fig 1: Enrichment plot: KEGG\_COMPLEMENT\_AND\_COAGULATION\_CASCADES      
 Profile of the Running ES Score & Positions of GeneSet Members on the Rank Ordered List

  

| SYMBOL | TITLE | RANK IN GENE LIST | RANK METRIC SCORE | RUNNING ES | CORE ENRICHMENT || 1 | CFI | complement factor I [Source:HGNC Symbol;Acc:HGNC:5394] | 455 | 0.680 | 0.0062 | No |
| 2 | C4A | complement C4A (Rodgers blood group) [Source:HGNC Symbol;Acc:HGNC:1323] | 632 | 0.611 | 0.0299 | No |
| 3 | C8B | complement C8 beta chain [Source:HGNC Symbol;Acc:HGNC:1353] | 1001 | 0.517 | 0.0329 | No |
| 4 | F8 | coagulation factor VIII [Source:HGNC Symbol;Acc:HGNC:3546] | 1292 | 0.459 | 0.0385 | No |
| 5 | CD55 | CD55 molecule (Cromer blood group) [Source:HGNC Symbol;Acc:HGNC:2665] | 1619 | 0.411 | 0.0382 | No |
| 6 | MBL2 | mannose binding lectin 2 [Source:HGNC Symbol;Acc:HGNC:6922] | 1856 | 0.379 | 0.0430 | No |
| 7 | C3AR1 | complement C3a receptor 1 [Source:HGNC Symbol;Acc:HGNC:1319] | 1933 | 0.368 | 0.0597 | No |
| 8 | CD59 | CD59 molecule (CD59 blood group) [Source:HGNC Symbol;Acc:HGNC:1689] | 2500 | 0.303 | 0.0342 | No |
| 9 | PROS1 | protein S [Source:HGNC Symbol;Acc:HGNC:9456] | 2511 | 0.302 | 0.0519 | No |
| 10 | KNG1 | kininogen 1 [Source:HGNC Symbol;Acc:HGNC:6383] | 2519 | 0.301 | 0.0698 | No |
| 11 | CR2 | complement C3d receptor 2 [Source:HGNC Symbol;Acc:HGNC:2336] | 2584 | 0.292 | 0.0827 | No |
| 12 | C3 | complement C3 [Source:HGNC Symbol;Acc:HGNC:1318] | 2764 | 0.273 | 0.0855 | No |
| 13 | C1QA | complement C1q A chain [Source:HGNC Symbol;Acc:HGNC:1241] | 2902 | 0.260 | 0.0908 | No |
| 14 | C1QB | complement C1q B chain [Source:HGNC Symbol;Acc:HGNC:1242] | 3365 | 0.215 | 0.0680 | No |
| 15 | SERPINA5 | serpin family A member 5 [Source:HGNC Symbol;Acc:HGNC:8723] | 3606 | 0.193 | 0.0611 | No |
| 16 | FGB | fibrinogen beta chain [Source:HGNC Symbol;Acc:HGNC:3662] | 3721 | 0.183 | 0.0635 | No |
| 17 | CFB | complement factor B [Source:HGNC Symbol;Acc:HGNC:1037] | 3743 | 0.182 | 0.0730 | No |
| 18 | C8A | complement C8 alpha chain [Source:HGNC Symbol;Acc:HGNC:1352] | 3918 | 0.163 | 0.0694 | No |
| 19 | C7 | complement C7 [Source:HGNC Symbol;Acc:HGNC:1346] | 4078 | 0.148 | 0.0661 | No |
| 20 | F5 | coagulation factor V [Source:HGNC Symbol;Acc:HGNC:3542] | 4223 | 0.135 | 0.0632 | No |
| 21 | C5 | complement C5 [Source:HGNC Symbol;Acc:HGNC:1331] | 4387 | 0.122 | 0.0579 | No |
| 22 | CD46 | CD46 molecule [Source:HGNC Symbol;Acc:HGNC:6953] | 4563 | 0.108 | 0.0509 | No |
| 23 | F2 | "coagulation factor II, thrombin [Source:HGNC Symbol;Acc:HGNC:3535]" | 4745 | 0.092 | 0.0424 | No |
| 24 | CFH | complement factor H [Source:HGNC Symbol;Acc:HGNC:4883] | 4964 | 0.075 | 0.0301 | No |
| 25 | F9 | coagulation factor IX [Source:HGNC Symbol;Acc:HGNC:3551] | 5185 | 0.057 | 0.0164 | No |
| 26 | F13B | coagulation factor XIII B chain [Source:HGNC Symbol;Acc:HGNC:3534] | 5265 | 0.050 | 0.0134 | No |
| 27 | CPB2 | carboxypeptidase B2 [Source:HGNC Symbol;Acc:HGNC:2300] | 5279 | 0.050 | 0.0154 | No |
| 28 | F10 | coagulation factor X [Source:HGNC Symbol;Acc:HGNC:3528] | 5293 | 0.048 | 0.0173 | No |
| 29 | C9 | complement C9 [Source:HGNC Symbol;Acc:HGNC:1358] | 5415 | 0.039 | 0.0103 | No |
| 30 | F11 | coagulation factor XI [Source:HGNC Symbol;Acc:HGNC:3529] | 5767 | 0.010 | -0.0164 | No |
| 31 | FGG | fibrinogen gamma chain [Source:HGNC Symbol;Acc:HGNC:3694] | 5997 | -0.004 | -0.0340 | No |
| 32 | F13A1 | coagulation factor XIII A chain [Source:HGNC Symbol;Acc:HGNC:3531] | 6203 | -0.021 | -0.0487 | No |
| 33 | BDKRB2 | bradykinin receptor B2 [Source:HGNC Symbol;Acc:HGNC:1030] | 6276 | -0.026 | -0.0527 | No |
| 34 | PLAUR | "plasminogen activator, urokinase receptor [Source:HGNC Symbol;Acc:HGNC:9053]" | 6362 | -0.032 | -0.0574 | No |
| 35 | F7 | coagulation factor VII [Source:HGNC Symbol;Acc:HGNC:3544] | 6745 | -0.059 | -0.0835 | No |
| 36 | SERPING1 | serpin family G member 1 [Source:HGNC Symbol;Acc:HGNC:1228] | 7286 | -0.101 | -0.1194 | No |
| 37 | C6 | complement C6 [Source:HGNC Symbol;Acc:HGNC:1339] | 7316 | -0.103 | -0.1153 | No |
| 38 | C1R | complement C1r [Source:HGNC Symbol;Acc:HGNC:1246] | 7716 | -0.135 | -0.1381 | Yes |
| 39 | PROC | "protein C, inactivator of coagulation factors Va and VIIIa [Source:HGNC Symbol;Acc:HGNC:9451]" | 7786 | -0.140 | -0.1349 | Yes |
| 40 | C4BPB | complement component 4 binding protein beta [Source:HGNC Symbol;Acc:HGNC:1328] | 7802 | -0.141 | -0.1274 | Yes |
| 41 | TFPI | tissue factor pathway inhibitor [Source:HGNC Symbol;Acc:HGNC:11760] | 7830 | -0.143 | -0.1208 | Yes |
| 42 | MASP1 | MBL associated serine protease 1 [Source:HGNC Symbol;Acc:HGNC:6901] | 8033 | -0.158 | -0.1268 | Yes |
| 43 | F3 | "coagulation factor III, tissue factor [Source:HGNC Symbol;Acc:HGNC:3541]" | 8152 | -0.167 | -0.1258 | Yes |
| 44 | F2R | coagulation factor II thrombin receptor [Source:HGNC Symbol;Acc:HGNC:3537] | 8447 | -0.190 | -0.1371 | Yes |
| 45 | SERPINC1 | serpin family C member 1 [Source:HGNC Symbol;Acc:HGNC:775] | 8590 | -0.200 | -0.1359 | Yes |
| 46 | SERPINF2 | serpin family F member 2 [Source:HGNC Symbol;Acc:HGNC:9075] | 8641 | -0.204 | -0.1273 | Yes |
| 47 | PLAT | "plasminogen activator, tissue type [Source:HGNC Symbol;Acc:HGNC:9051]" | 8689 | -0.208 | -0.1182 | Yes |
| 48 | C8G | complement C8 gamma chain [Source:HGNC Symbol;Acc:HGNC:1354] | 8871 | -0.223 | -0.1186 | Yes |
| 49 | A2M | alpha-2-macroglobulin [Source:HGNC Symbol;Acc:HGNC:7] | 8876 | -0.223 | -0.1053 | Yes |
| 50 | FGA | fibrinogen alpha chain [Source:HGNC Symbol;Acc:HGNC:3661] | 8880 | -0.223 | -0.0919 | Yes |
| 51 | CFD | complement factor D [Source:HGNC Symbol;Acc:HGNC:2771] | 9154 | -0.244 | -0.0982 | Yes |
| 52 | VWF | von Willebrand factor [Source:HGNC Symbol;Acc:HGNC:12726] | 9180 | -0.246 | -0.0850 | Yes |
| 53 | C4BPA | complement component 4 binding protein alpha [Source:HGNC Symbol;Acc:HGNC:1325] | 9505 | -0.274 | -0.0935 | Yes |
| 54 | SERPINA1 | serpin family A member 1 [Source:HGNC Symbol;Acc:HGNC:8941] | 9837 | -0.305 | -0.1006 | Yes |
| 55 | KLKB1 | kallikrein B1 [Source:HGNC Symbol;Acc:HGNC:6371] | 9862 | -0.308 | -0.0836 | Yes |
| 56 | C1S | complement C1s [Source:HGNC Symbol;Acc:HGNC:1247] | 10012 | -0.323 | -0.0754 | Yes |
| 57 | SERPINE1 | serpin family E member 1 [Source:HGNC Symbol;Acc:HGNC:8583] | 10284 | -0.349 | -0.0752 | Yes |
| 58 | PLAU | "plasminogen activator, urokinase [Source:HGNC Symbol;Acc:HGNC:9052]" | 10746 | -0.398 | -0.0867 | Yes |
| 59 | PLG | plasminogen [Source:HGNC Symbol;Acc:HGNC:9071] | 10939 | -0.423 | -0.0757 | Yes |
| 60 | BDKRB1 | bradykinin receptor B1 [Source:HGNC Symbol;Acc:HGNC:1029] | 10951 | -0.424 | -0.0505 | Yes |
| 61 | C5AR1 | complement C5a receptor 1 [Source:HGNC Symbol;Acc:HGNC:1338] | 10978 | -0.427 | -0.0264 | Yes |
| 62 | MASP2 | MBL associated serine protease 2 [Source:HGNC Symbol;Acc:HGNC:6902] | 11001 | -0.430 | -0.0018 | Yes |
| 63 | CR1 | complement C3b/C4b receptor 1 (Knops blood group) [Source:HGNC Symbol;Acc:HGNC:2334] | 11376 | -0.483 | -0.0013 | Yes |
| 64 | SERPIND1 | serpin family D member 1 [Source:HGNC Symbol;Acc:HGNC:4838] | 11938 | -0.580 | -0.0095 | Yes |
| 65 | THBD | thrombomodulin [Source:HGNC Symbol;Acc:HGNC:11784] | 12014 | -0.596 | 0.0212 | Yes |
| 66 | F12 | coagulation factor XII [Source:HGNC Symbol;Acc:HGNC:3530] | 12565 | -0.780 | 0.0261 | Yes |
Table: GSEA details [plain text format]

  

Fig 2: KEGG\_COMPLEMENT\_AND\_COAGULATION\_CASCADES      
 Blue-Pink O' Gram in the Space of the Analyzed GeneSet

  

Fig 3: KEGG\_COMPLEMENT\_AND\_COAGULATION\_CASCADES: Random ES distribution      
 Gene set null distribution of ES for **KEGG\_COMPLEMENT\_AND\_COAGULATION\_CASCADES**
